# A Mobile Clinical Decision Support Tool for Pediatric Cardiovascular Risk-Reduction Clinical Practice Guidelines: Development and Description

**DOI:** 10.2196/mhealth.6291

**Published:** 2017-03-07

**Authors:** Robert D Furberg, Pamela Williams, Jacqueline Bagwell, Kenneth LaBresh

**Affiliations:** ^1^ Digital Health and Clinical Informatics RTI International Research Triangle Park, NC United States; ^2^ Center for Communication Science RTI International Research Triangle Park, NC United States; ^3^ Division for Health Services and Social Policy Research RTI International Research Triangle Park, NC United States

**Keywords:** pediatrics, cardiovascular risk reduction, mHealth, clinical decision support, clinical practice guidelines

## Abstract

**Background:**

Widespread application of research findings to improve patient outcomes remains inadequate, and failure to routinely translate research findings into daily clinical practice is a major barrier for the implementation of any evidence-based guideline. Strategies to increase guideline uptake in primary care pediatric practices and to facilitate adherence to recommendations are required.

**Objective:**

Our objective was to operationalize the US National Heart, Lung, and Blood Institute’s *Integrated Guidelines for Cardiovascular Health and Risk Reduction in Children and Adolescents* into a mobile clinical decision support (CDS) system for healthcare providers, and to describe the process development and outcomes.

**Methods:**

To overcome the difficulty of translating clinical practice guidelines into a computable form that can be used by a CDS system, we used a multilayer framework to convert the evidence synthesis into executable knowledge. We used an iterative process of design, testing, and revision through each step in the translation of the guidelines for use in a CDS tool to support the development of 4 validated modules: an integrated risk assessment; a blood pressure calculator; a body mass index calculator; and a lipid management instrument.

**Results:**

The iterative revision process identified several opportunities to improve the CDS tool. Operationalizing the integrated guideline identified numerous areas in which the guideline was vague or incorrect and required more explicit operationalization. Iterative revisions led to workable solutions to problems and understanding of the limitations of the tool.

**Conclusions:**

The process and experiences described provide a model for other mobile CDS systems that translate written clinical practice guidelines into actionable, real-time clinical recommendations.

## Introduction

Cardiovascular disease (CVD) is the leading cause of death in the United States [1] and accounts for over US $108 billion in health care expenditures annually [2]. While appreciable heart disease in children and adolescents is rare, health behaviors, risk factors, and exposures beginning in childhood can accelerate the development of atherosclerosis. Evidence that most CVD is preventable led to the development of primary prevention guidelines for adults [3], and there has since been increasing evidence that risk-reduction strategies may also delay progression toward clinical disease among younger populations [4].

In 2006, the US National Heart, Lung, and Blood Institute (NHLBI) established an Expert Panel to develop comprehensive evidence-based guidelines addressing the known risk factors for CVD among the pediatric population, including family history, age, sex, nutrition and diet, physical inactivity, tobacco exposure, blood pressure (BP), lipids, overweight and obesity, diabetes mellitus, predisposing conditions, metabolic syndrome, inflammatory markers, and perinatal factors. The guidelines are intended for use by a broad audience of pediatric care providers—such as pediatricians, family practitioners, nurses, nurse practitioners, physician assistants, and registered dietitians—to assist in the promotion of cardiovascular health and the identification and management of specific risk factors from infancy into young adult life, with an integrated format to address the 14 major risk factors simultaneously.

In practice, pediatric care providers are credible messengers of health information, and the guideline recommendations are intended to accommodate the delivery of health messages to patients and their families by all members of the care team. The Expert Panel identified childhood health maintenance visits as an ideal context for engaging patients to increase awareness, initiate risk-reduction strategies, and promote cardiovascular health. The NHLBI guidelines provide recommendations that are specific to the age and developmental stage of the patient, considering not only the relation of age to disease expression, but also the ability of the patient and family to understand and implement medical advice. Management algorithms provide staged care recommendations for risk reduction within the pediatric care setting and identify risk factor levels that require referral from the primary care setting to a specialist. The guidelines also identify specific medical conditions, including diabetes and chronic kidney disease, associated with increased risk for accelerated atherosclerosis and provide recommendations for ongoing management of children and adolescents with these diagnoses. The report, entitled the *Integrated Guidelines for Cardiovascular Health and Risk Reduction in Children and Adolescents*, is more than 400 pages and it is available as a searchable online version or can be downloaded as a PDF from NHLBI’s public website [5]. A guideline summary report was published in a special issue of the journal *Pediatrics* in 2011 [6].

Failure to routinely translate research findings into daily clinical practice is a major barrier for the implementation of any evidence-based guideline and is related to barriers of knowledge, attitudes, and clinical systems [7]. In a seminal review on guideline uptake, Balas [8] suggested that it takes an average of 17 years for 14% of the original research to be integrated into clinical practice. Although methods used to develop evidence-based guidelines have improved [9,10], the widespread application of research findings to improve patient outcomes remains inadequate [7,11,12]. Strategies to improve uptake will need to enhance clinician knowledge and support confidence that guidelines can be implemented (self-efficacy) and integrated into clinical workflow.

The adoption of electronic health records in primary care, as enabled by the Health Information Technology for Economic and Clinical Health (HITECH) Act and the pursuit of satisfying meaningful use objectives, has not inhibited the use of clinical decision support (CDS) tools in practice. Defined by Osheroff et al [13] as “providing clinicians or patients with computer-generated clinical knowledge and patient-related information, intelligently filtered or presented at appropriate times to enhance patient care,” these resources may hold the potential to achieve gains in clinical performance, narrow gaps between knowledge and practice, and improve safety [14]. Systematic reviews of CDS systems have demonstrated effectiveness in improving clinician practice and guideline adherence [15]. A comprehensive evaluation of systematic reviews by Jaspers et al [16] found strong evidence that CDS systems improved practitioner performance in this regard.

From the outset, implementation of the *Integrated Guidelines for Cardiovascular Health and Risk Reduction in Children and Adolescents* was expected to pose a variety of challenges that were unique to the composition of the content. These issues include (1) the complexity and integrated nature of risk assessment and management, (2) the volume of information provided in the final report, (3) the establishment of prevention as a clinical priority among a nondiseased target audience, and (4) the delivery of multifaceted recommendations within the time-constrained context of the pediatric primary care setting.

Evidence-based clinical practice guidelines represent substantial effort on the part of their authors and considerable financial support to sustain years-long review and synthesis of scientific evidence. Despite the investment to rigorously develop guidelines, implementation efforts have often been less well supported and often unsuccessful in improving care, and other efforts have reported outright implementation failures [17-20]. This suggests the need for a more rigorous approach to the development of CDS systems for guideline implementation.

Consequently, in this paper we describe the process and outcomes of a project to operationalize the NHLBI guidelines into a mobile CDS system and provide multimedia artifacts associated with each step in the conversion process. The CDS was made available to pediatric primary care providers through an intervention called *Young Hearts, Strong Starts*; the methods and results from a cluster randomized trial of guideline adoption using quality improvement techniques to integrate the CDS into clinical workflows are described elsewhere by LaBresh et al [21,22]. At the time of publication, the CDS app remains publicly available from NHLBI at no cost in iTunes [23].

## Methods

Evidence-based clinical practice guidelines are defined by the Institute of Medicine as “systematically developed statements intended to assist practitioner and patient decision-making about appropriate healthcare for specific clinical circumstances” [24]. However, clinical practice guidelines are not routinely written in a format suitable for conversion into a computable form that can be used by a CDS system, necessitating use of a multilayer framework [20] for incrementally structuring guideline recommendations and converting the evidence synthesis into executable knowledge for implementation. Other layered knowledge representations that have been used in the development of CDS resources include the digital electronic guidelines library framework [25] and the guideline elements model [26]; however, such approaches require the organization of information in an explicit flow of events. Given the asynchronous nature of the ongoing assessment of pediatric cardiovascular health, we selected an alternative framework that emphasized modeling of unsequenced clinical decisions. This approach enabled development of a CDS tool appropriate for a greater variety of point-in-time assessments, rather than forcing a complete physical assessment and data entry in each session. The multilayered framework provides a reproducible approach to the progressive conversion from scientific material to technical artifacts and code base. Each layer (ie, unstructured, semistructured, structured, and executable) serves a different purpose and leverages the expertise of different roles required to implement the CDS. Different roles are involved at each step, including business stakeholders, analysts, architects, and programmers.

We used an iterative approach to the design, testing, and revision of material through each step in the translation to support the development of 4 validated modules: an integrated screener for risk assessment, a body mass index (BMI) calculator, a BP calculator, and a lipid assessment and management instrument. This information architecture was informed initially by the structure of the guideline content and reinforced by stakeholder input on clinical decision making during an initial well-child visit and subsequent follow-up to address specific findings. Our approach is described below in more detail. Multimedia Appendix 1 illustrates the progressive conversion of the evidence-based recommendations for management of overweight and obesity. Semistructured guideline content is included as Multimedia Appendix 2; structured guideline content is included as Multimedia Appendix 3; and executable guideline content for the calculator modules is included as Multimedia Appendix 4.

### Unstructured Layer

This layer consists of the narrative and figures of the comprehensive evidence-based guidelines documents published by NHLBI to address the known risk factors for CVD, including family history, age, sex, nutrition and diet, physical inactivity, tobacco exposure, BP, lipids, overweight and obesity, diabetes mellitus, predisposing conditions, metabolic syndrome, inflammatory markers, and perinatal factors. Our initial effort focused on deconstructing the guidelines into a tabular framework of triggers (eg, age, elevated BMI), recommendations, and supportive actions, as organized by known risk factor. We then used the deconstructed guidelines as a roadmap to articulate the business logic to document how a patient’s presentation to a clinician would qualify the patient into each known risk factor category. This approach to structuring the guideline content also enabled authoring a series of use cases for potential clinical encounters, beginning with an integrated risk assessment, then progressing to follow-up encounters based on the recommended treatment and follow-up for the mitigation of specific risk factors.

### Semistructured Layer

In this layer, we added structure to the guidelines. The core organizing concept for the knowledge is a recommendation that has been triggered by the presence of a specific risk factor. From this layer onward, the emphasis is on processing within the CDS, and the recommendations excluded any statements that were not patient specific. Because domain experts are expected to be the primary authors of knowledge presented in this layer, we engaged the NHLBI Expert Panel Chair responsible for authoring the guidelines to support ongoing review of the conversion. This stage yielded a complete guideline framework in a tabular format, a matrix to illustrate how each variable triggers each recommendation, and a summary narrative of use cases.

### Structured Layer

In this layer, the knowledge is specified with sufficient structure so as to make it computable and precise. This knowledge is independent of the implementation in a particular type of CDS tool or of the workflow in a particular clinical setting; however, it serves to formally define all the data elements and logic required to do so. The objective of this layer is to communicate the knowledge in the guidelines to CDS implementers to inform development of executable code. We relied heavily on a collaborative team of clinical informaticists and developers to support the translation of clinical domain requirements for computer implementation.

### Executable Layer

In this layer, the knowledge is structured for use within a specific type of CDS system. This knowledge is less likely to be sharable because often it includes elements that apply only to that setting; for example, local codes for data items, details of local clinical services, and idiosyncrasies of how the end user interacts with the system. Our implementation was centered on the Personal Health Intervention Toolkit mobile technical framework that has been described elsewhere [27].

### Data Model

Based on the multilayer framework, we created narrative use cases based on models of the clinical decision-making process, in addition to class diagrams in the unified modeling language and equivalent XML schemas, for representing guideline recommendations in the semistructured and the structured recommendation layers. We did not create a knowledge representation model for the unstructured layer because that comprises the free-form published guideline document. Further modeling of the executable layer is of limited utility given the specific implementation context that was used for this project.

### Validation

We subjected the CDS tool to rigorous expert review throughout all stages of the conversion process. Once compiled, the app was unit tested and subjected to formal software quality assurance testing of the fully integrated tool. We developed over 400 single-expression test cases to test individual CDS rules. These cases tested the output of a specific recommendation based on data element inputs defined in the rules. For these tests, values at, below, and above the rule boundary were tested.

We developed over 70 scenarios to exercise the system under realistic conditions and to test data element inputs across multiple domains and thresholds. In addition to tests that verified the correct recommendation, code was generated from each rule and each rule was tested to ensure it resulted in only its specified recommendation output. We tested each numeric value boundary by cases that included the thresholds and one or more values of the unit in whole numbers above or below the threshold. Values were tested in each tool and the integrated assessment regardless of duplication (ie, weight boundary tests were completed for each tool and component).

## Results

We used an iterative process of design, testing, and revision through each layer of conversion, resulting in the development of a management instrument and 4 validated modules: an integrated assessment, a BMI calculator, a BP calculator, and a lipid assessment. Formatting content in each successive layer enabled expert review of the variables, triggers, and recommendation copy. On the basis of the layered representations and conversion process, we refined detailed recommendations for ease of presentation on a mobile device and developed logic to eliminate display of duplicative recommendations or supportive actions. In addition, we identified a misalignment of categories used for age-based recommendations and overlap in the NHLBI guidelines for individuals in the infant to 3-year-old and 3- to 11-year-old groups. The results of the formative user-centered design and user experience testing [28], and implementation protocol and results are described elsewhere [21,22].

**Figure 1:**
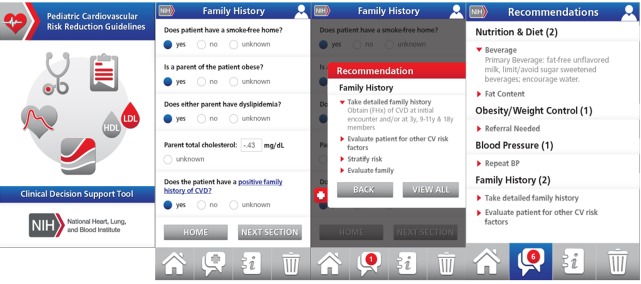
Pediatric cardiovascular (CV) risk-reduction clinical decision support app home screen and modules. BP: blood pressure; CVD: cardiovascular disease; FHx: family history; HDL: high-density lipoprotein; LDL: low-density lipoprotein; NIH: National Institutes of Health.

When the user first enters the app on its home screen, there is the option of entering 1 of the 4 modules noted above (see Figure 1). Along the bottom of the screen, the user also has the option to toggle between 4 screens: the home screen; a recommendations screen (based on the data entered from each of the modules); a guidelines screen (with data entry instructions; a link to the guidelines website; copyright, disclaimer, and terms of use; and contact information to provide feedback on the app); and a “trashcan” screen that offers the option to clear the current patient’s data. Below we describe the features of each of the 4 modules.

### Integrated Risk Assessment Screener

This module incorporates assessments of BMI, BP, and other risk factors, and then provides users with a patient summary and US National Institutes of Health (NIH) recommendations based on the patient’s risk factor information.

#### Body Mass Index

First, the user is asked to enter the patient’s date of birth, and the app automatically calculates the age. Then the user enters the patient’s sex, height, and weight, and the app automatically computes the BMI and the corresponding BMI-for-age percentile based on the US Centers for Disease Control and Prevention’s BMI-for-age growth chart [29] and indicates if the patient is overweight or obese. Finally, the user is asked to categorize the change in BMI as stable, improvement, increase, excessive increase, no improvement, or unknown.

#### Blood Pressure

The user is asked to enter the patient’s systolic and diastolic BP, and if the user hits a designated button, the app calculates the BP percentile based on the NHLBI’s BP tables for children and adolescents [30].

#### Other Risk Factors

The user is then asked to indicate whether the patient has any of the following conditions: hypertension, dyslipidemia, type 1 diabetes mellitus, type 2 diabetes mellitus, none of these, or unknown. If so, the user is asked to categorize the change in each of the condition as improving, not improving, or unknown. Then the user is asked whether the patient has any other high or moderate risk conditions, with response options of yes, no, or unknown. “Risk conditions” is underlined and shown in a different color, a technique commonly used on the Internet to indicate that the user can click on a link to get the definition. The user is then asked to categorize the patient’s physical activity level as sedentary, moderate to vigorous, vigorous, or unknown, and whether this is a decrease in physical activity level (response options: no, yes, or unknown). The user is then asked whether the patient currently smokes (yes, no, or unknown) and whether the patient has a smoke-free home (yes, no, or unknown). Then the user is asked about parental risk factors; namely, whether either parent is obese (yes, no, or unknown), whether either parent has dyslipidemia (yes, no, or unknown), the mother’s total cholesterol (or unknown), and the father’s total cholesterol (or unknown). Finally, the user is asked whether the patient has a positive family history of CVD (yes, no, or unknown). “Positive family history of CVD” is underlined and displayed in a different color indicating that the user can click on this link to get the definition.

When a user indicates that data entry is complete, the app automatically moves to the recommendations screen showing a patient summary of the data entered. Then, based on the patient’s risk factors, the user is shown the relevant NIH recommendations, including the evidence grade (A-F), related to family history, nutrition and diet, physical activity, tobacco exposure, lipids, and overweight and obesity, as well as supportive actions to take or more information, in some instances.

### Body Mass Index Calculator

If the user has already entered information in the integrated assessment screener, then the app displays the relevant previously entered data in this module. The user simply needs to click on the Calculate BMI button to see the BMI, BMI-for-age percentile, and indication of whether the patient is overweight or obese. When a user indicates that data entry is complete, as with the integrated assessment screener, the app automatically presents the recommendations screen showing a patient summary of the data entered and the relevant NIH recommendations.

In some instances, the user may only be interested in using the BMI calculator. If so, the user is asked to enter the same information described above under the BMI portion of the integrated assessment screener (ie, patient’s date of birth, sex, height, and weight) to obtain the BMI information, a patient summary of the data entered, and the recommendations.

### Blood Pressure Calculator

As with the BMI calculator, if the user has already entered BP information in the integrated assessment screener, then the app will display the previously entered data in this module. In instances where the user is only interested in using the BP calculator, they are asked to enter the same information described above under the BP portion of the integrated assessment screener (ie, patient’s systolic and diastolic BP). All users are then asked to enter additional BP data in this module: how the BP was measured at this visit (with response options of auscultation, oscillometry, or unknown) and up to 3 BP readings. Then the user clicks on a designated button and the app calculates the average systolic and diastolic BP values, as well as the systolic and diastolic BP percentiles. As with the other modules, when a user indicates that data entry is complete, the app automatically moves to the recommendations screen showing a patient summary of the data entered and the relevant recommendations.

### Lipid Assessment

As with the BMI and BP calculators, if the user has already entered information in other modules, then the app will display the relevant previously entered data in this module (ie, patient’s date of birth, sex, height, and weight). In instances where the user is only interested in using the lipid assessment, they are asked to enter this information. Then the user simply needs to click on the Calculate BMI button to see the BMI, BMI-for-age percentile, and indication of whether the patient is overweight or obese. All users are then asked to enter the type of sample that was drawn (with response options of fasting, nonfasting, or unknown). As with the other modules, when a user indicates that data entry is complete, the app automatically moves to the recommendations screen showing a patient summary of the data entered and the recommendations.

## Discussion

### Principal Findings

The NHLBI integrated guideline is long and extremely complex as a result of the desire to put all relevant cardiovascular risk-reduction recommendations in a single document. Because we know that guideline complexity is a barrier to guideline use [7], the CDS tool provided a means to integrate a large amount of information and recommended processes into a single handheld app. Our app was built for mobile devices to allow for greater uptake and ease of use. Much has been written about the complexity of electronic medical records that in some cases detract from clinician-patient interactions [31]. This CDS tool was not designed to support documentation of the patient encounter, but rather to enable precise, contextualized information retrieval from the NHLBI integrated guideline to the clinician during a well-child or follow-up visit. During the clinical implementation phase, the mobile version was considered far less obtrusive and was available for use by any clinician or staff member with a mobile phone or tablet, thus enhancing clinician uptake [21,22].

Cabana et al [7] noted that guideline uptake is primarily limited by 3 factors: knowledge, attitudes, and behavior (systems). The CDS tool addresses knowledge by providing brief, relevant information based on the risk factors present in the patient being seen and consequently provides only the relevant knowledge needed at the point in the care process when risk is being assessed and recommendations are being made. In the cluster randomized trial [21,22], risk such as elevated BP could be assessed by the medical assistant using the tool without the need to consult multiple tables to determine BP percentiles, which would speed up the process and allow physicians and nurse practitioners to delegate the responsibility to staff early in the process. This allows the clinician to focus on counseling the patient and their family to adopt healthy lifestyle changes. The result was an increase in the percentage of patients for whom BP percentile was determined from 0.2% at baseline to 61.3% at 1 year after implementation, with no change in the control group, who did not have the CDS tool. Consequently, the CDS resulted in increased practice efficiency, with medical assistants performing important clinical assessments with the CDS, and allowing more time for clinicians to help patients and their families address needed lifestyle changes or, in some patients, the use of medications.

### Conclusion

To overcome the challenges of translating clinical practice guidelines into a computable form for use in a CDS clinical system, we used a multilayer framework to convert the evidence synthesis into executable knowledge. We used an iterative process of design, testing, and revision through each step in the translation of the guidelines for use in a CDS tool to support the development of 4 validated modules: an integrated risk assessment; a BMI calculator; a BP calculator; and a lipid management instrument. The process and experiences described here provide a model for other mobile CDS systems that translate written clinical practice guidelines into actionable, real-time clinical recommendations.

## Appendix

Multimedia Appendix 1 Progressive conversion of the evidence-based recommendations for management of overweight and obesity.

Multimedia Appendix 2 Semistructured guideline content.

Multimedia Appendix 3 Structured guideline content.

Multimedia Appendix 4 Executable guideline content.

## Abbreviations

BMI: body mass index
BP: blood pressure
CDS: clinical decision support
CVD: cardiovascular disease
HITECH: Health Information Technology for Economic and Clinical Health
NHLBI: National Heart, Lung, and Blood Institute
NIH: National Institutes of Health
