# Supplementary material for: A Mobile Clinical Decision Support Tool for Pediatric Cardiovascular Risk-Reduction Clinical Practice Guidelines: Development and Description
Source: JMIR Mhealth Uhealth. 2017 Mar 7;5(3):e29. doi: 10.2196/mhealth.6291 (PMC5378042; doi:10.2196/mhealth.6291)
Supplement: Multimedia Appendix 1 [file mhealth_v5i3e29_app1.pptx]

## Slide 1
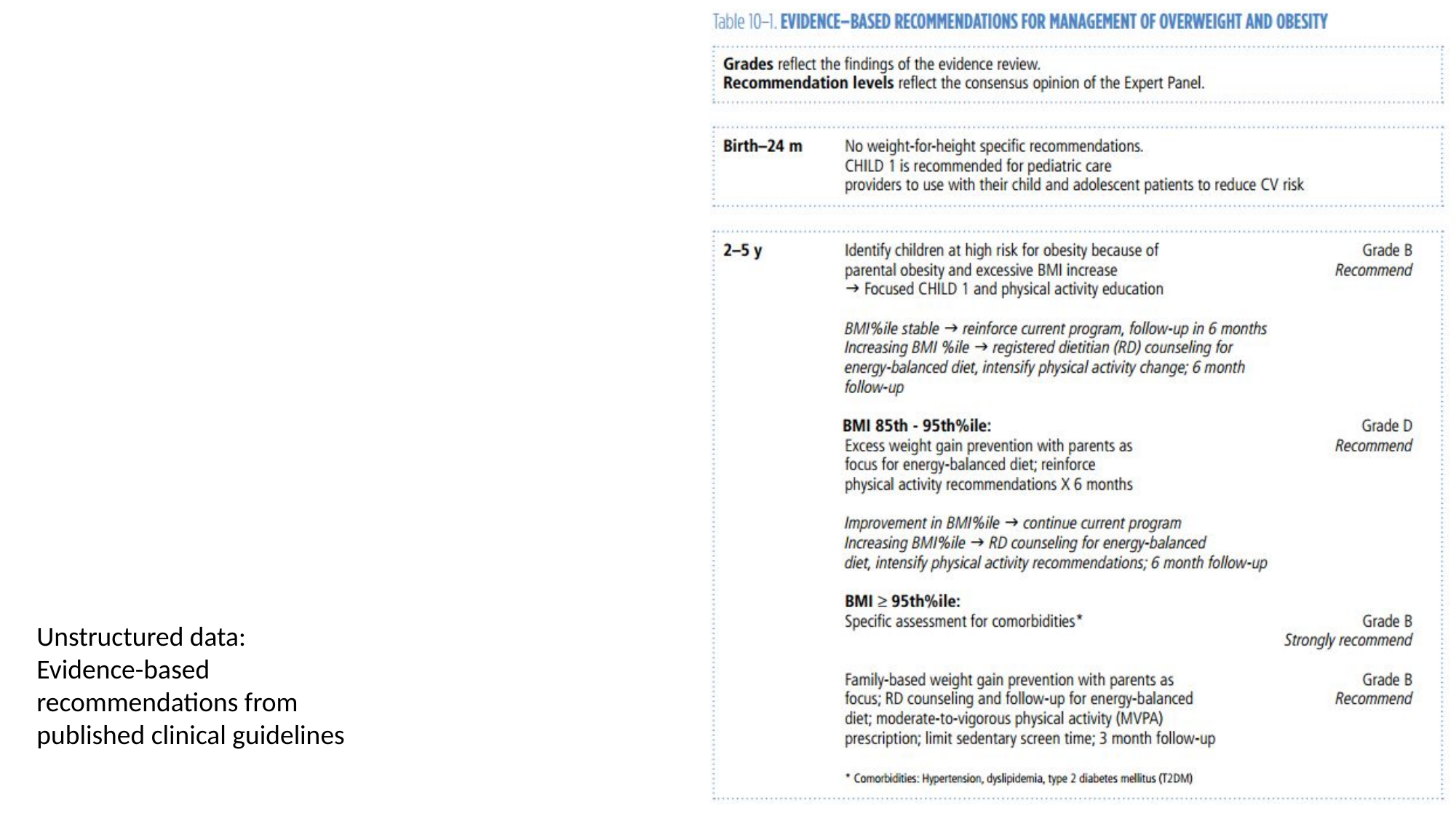

Unstructured data: Evidence-based recommendations from published clinical guidelines

## Slide 2
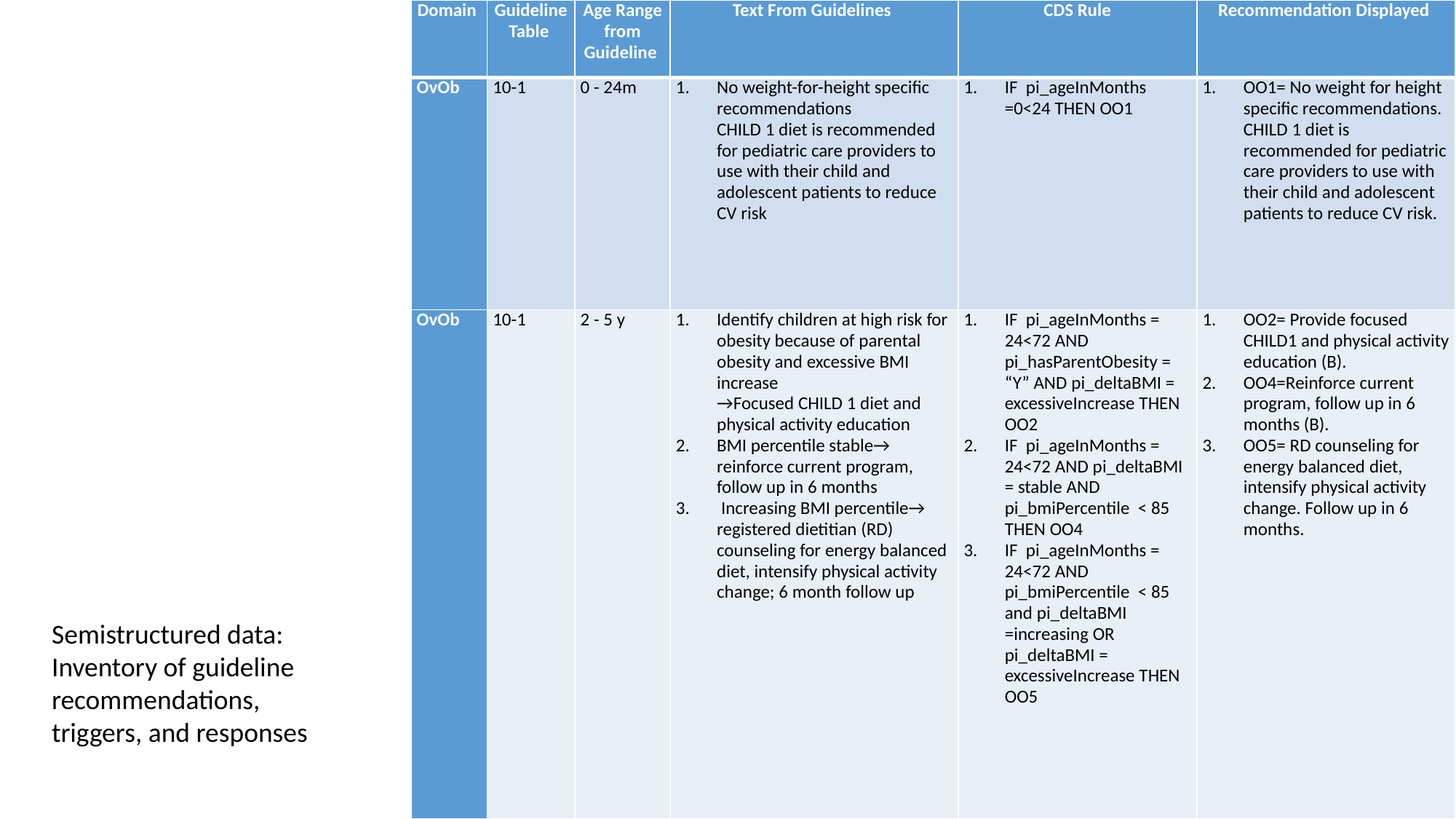

| Domain | Guideline Table | Age Range from Guideline | Text From Guidelines | CDS Rule | Recommendation Displayed |
| --- | --- | --- | --- | --- | --- |
| OvOb | 10-1 | 0 - 24m | No weight-for-height specific recommendations CHILD 1 diet is recommended for pediatric care providers to use with their child and adolescent patients to reduce CV risk | IF pi\_ageInMonths =0<24 THEN OO1 | OO1= No weight for height specific recommendations. CHILD 1 diet is recommended for pediatric care providers to use with their child and adolescent patients to reduce CV risk. |
| OvOb | 10-1 | 2 - 5 y | Identify children at high risk for obesity because of parental obesity and excessive BMI increase →Focused CHILD 1 diet and physical activity education BMI percentile stable→ reinforce current program, follow up in 6 months Increasing BMI percentile→ registered dietitian (RD) counseling for energy balanced diet, intensify physical activity change; 6 month follow up | IF pi\_ageInMonths = 24<72 AND pi\_hasParentObesity = “Y” AND pi\_deltaBMI = excessiveIncrease THEN OO2 IF pi\_ageInMonths = 24<72 AND pi\_deltaBMI = stable AND pi\_bmiPercentile < 85 THEN OO4 IF pi\_ageInMonths = 24<72 AND pi\_bmiPercentile < 85 and pi\_deltaBMI =increasing OR pi\_deltaBMI = excessiveIncrease THEN OO5 | OO2= Provide focused CHILD1 and physical activity education (B). OO4=Reinforce current program, follow up in 6 months (B). OO5= RD counseling for energy balanced diet, intensify physical activity change. Follow up in 6 months. |
Semistructured data: Inventory of guideline recommendations, triggers, and responses

## Slide 3
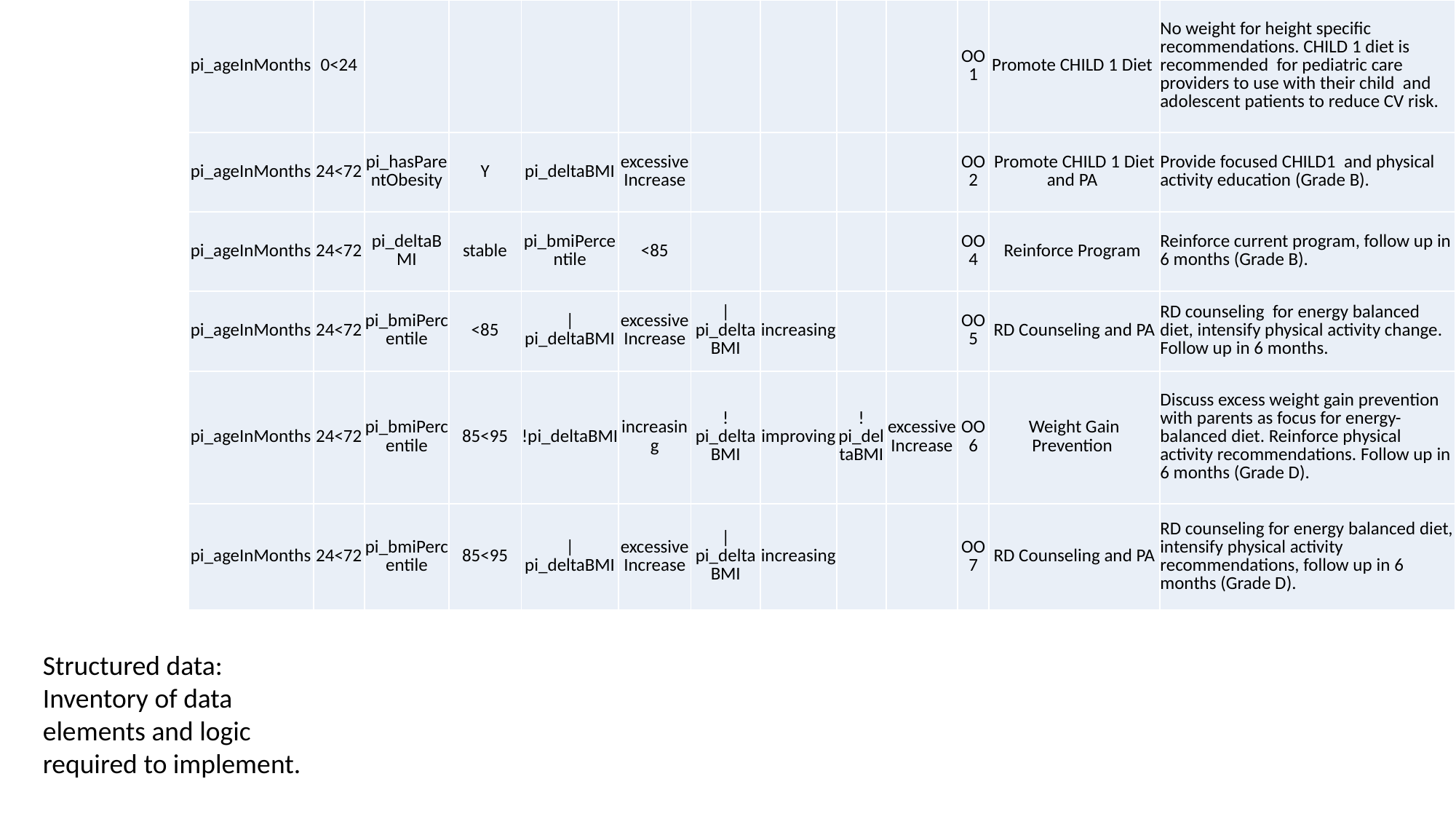

| pi\_ageInMonths | 0<24 | | | | | | | | | OO1 | Promote CHILD 1 Diet | No weight for height specific recommendations. CHILD 1 diet is recommended for pediatric care providers to use with their child and adolescent patients to reduce CV risk. |
| --- | --- | --- | --- | --- | --- | --- | --- | --- | --- | --- | --- | --- |
| pi\_ageInMonths | 24<72 | pi\_hasParentObesity | Y | pi\_deltaBMI | excessiveIncrease | | | | | OO2 | Promote CHILD 1 Diet and PA | Provide focused CHILD1 and physical activity education (Grade B). |
| pi\_ageInMonths | 24<72 | pi\_deltaBMI | stable | pi\_bmiPercentile | <85 | | | | | OO4 | Reinforce Program | Reinforce current program, follow up in 6 months (Grade B). |
| pi\_ageInMonths | 24<72 | pi\_bmiPercentile | <85 | |pi\_deltaBMI | excessiveIncrease | |pi\_deltaBMI | increasing | | | OO5 | RD Counseling and PA | RD counseling for energy balanced diet, intensify physical activity change. Follow up in 6 months. |
| pi\_ageInMonths | 24<72 | pi\_bmiPercentile | 85<95 | !pi\_deltaBMI | increasing | !pi\_deltaBMI | improving | !pi\_deltaBMI | excessiveIncrease | OO6 | Weight Gain Prevention | Discuss excess weight gain prevention with parents as focus for energy-balanced diet. Reinforce physical activity recommendations. Follow up in 6 months (Grade D). |
| pi\_ageInMonths | 24<72 | pi\_bmiPercentile | 85<95 | |pi\_deltaBMI | excessiveIncrease | |pi\_deltaBMI | increasing | | | OO7 | RD Counseling and PA | RD counseling for energy balanced diet, intensify physical activity recommendations, follow up in 6 months (Grade D). |
Structured data: Inventory of data elements and logic required to implement.

## Slide 4
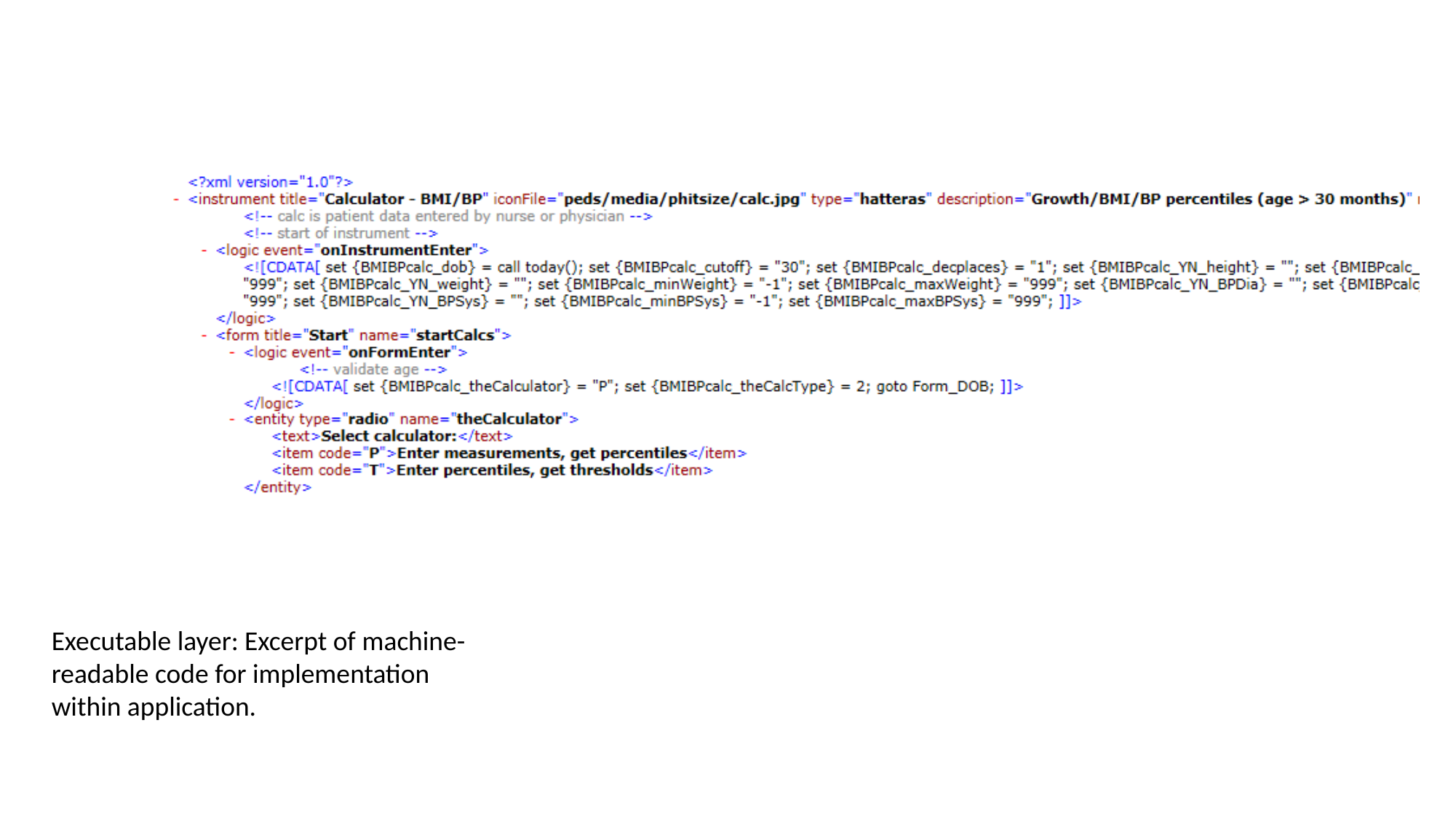

Executable layer: Excerpt of machine-readable code for implementation within application.
