# Supplementary material for: A Mobile Clinical Decision Support Tool for Pediatric Cardiovascular Risk-Reduction Clinical Practice Guidelines: Development and Description
Source: JMIR Mhealth Uhealth. 2017 Mar 7;5(3):e29. doi: 10.2196/mhealth.6291 (PMC5378042; doi:10.2196/mhealth.6291)
Supplement: Multimedia Appendix 2 [file mhealth_v5i3e29_app2.pdf]

Domain	Guideline Table	Age Range from Guideline	Text From Guidelines	CDS Rule	Recommendation Displayed
OvOb	10-1	0 - 24m	1. No weight-for-height specific recommendations<br>CHILD 1 diet is recommended for pediatric care providers to use with their child and adolescent patients to reduce CV risk	1. IF pi_ageInMonths =0<24 THEN OO1	1. OO1= No weight for height specific recommendations. CHILD 1 diet is recommended for pediatric care providers to use with their child and adolescent patients to reduce CV risk.
OvOb	10-1	2 - 5 y	1. Identify children at high risk for obesity because of parental obesity and excessive BMI increase →Focused CHILD 1 diet and physical activity education<br>2. BMI percentile stable→ reinforce current program, follow up in 6 months<br>3. Increasing BMI percentile→ registered dietitian (RD) counseling for energy balanced diet, intensify physical activity change; 6 month follow up	1. IF pi_ageInMonths = 24<72 AND pi_hasParentObesity = “Y” AND pi_deltaBMI = excessiveIncrease THEN OO2<br>2. IF pi_ageInMonths = 24<72 AND pi_deltaBMI = stable AND pi_bmiPercentile < 85 THEN OO4<br>3. IF pi_ageInMonths = 24<72 AND pi_bmiPercentile < 85 and pi_deltaBMI =increasing OR pi_deltaBMI = excessiveIncrease THEN OO5	1. OO2= Provide focused CHILD1 and physical activity education (B).<br>2. OO4=Reinforce current program, follow up in 6 months (B).<br>3. OO5= RD counseling for energy balanced diet, intensify physical activity change. Follow up in 6 months.

Domain	Guideline Table	Age Range from Guideline	Text From Guidelines	CDS Rule	Recommendation Displayed
OvOb	10-1	2 - 5 y	1. BMI 85th-95th percentile: Excess weight gain prevention with parents as focus for energy- balanced diet; reinforce physical activity recommendations X 6 months<br>2. Improvement in BMI percentile → continue current program<br>3. Increasing BMI percentile → RD counseling for energy-balanced diet; intensify physical activity recommendations; 6 month follow up	1. IF pi_ageInMonths 24<72 AND pi_bmiPercentile 85<95 AND pi_deltaBMI ≠ increasing AND pi_deltaBMI≠ improving AND pi_deltaBMI ≠ excessiveIncrease THEN OO6<br>2. IF pi_ageInMonths= 24<72 AND pi_bmiPercentile= 85<95 AND pi_deltaBMI = improving THEN 008<br>3. IF pi_ageInMonths= 24<72 AND pi_bmiPercentile= 85<95 AND pi_deltaBMI= excessiveIncrease OR pi_deltaBMI= Increasing THEN OO7	1. OO6=Discuss excess weight gain prevention with parents as focus for energy-balanced diet. Reinforce physical activity recommendations. Follow up in 6 months (D).<br>2. OO8= Continue current program.<br>3. OO7=RD counseling for energy balanced diet, intensify physical activity recommendations; follow up in 6 months (D).

Domain	Guideline Table	Age Range from Guideline	Text From Guidelines	CDS Rule	Recommendation Displayed
OvOb	10-1	2 - 5 y	1. BMI ≥ 95th percentile: Specific assessment for comorbidities* Family-based weight gain prevention with parents as focus; RD counseling and follow up for energy-balanced diet; moderate-to-vigorous physical activity (MVPA) prescription; limit sedentary screen time; 3 month followup	1. IF pi_ageInMonths = 24<72 AND pi_bmiPercentile= >=95 THEN OO9	1. OO9= Assess for comorbidities. Family-based weight gain prevention with parents as focus; RD counseling and follow-up for energy-balanced diet; moderate to vigorous physical activity (MVPA) prescription; limit sedentary screen time. Follow up in 3 months (B).

Domain	Guideline Table	Age Range from Guideline	Text From Guidelines	CDS Rule	Recommendation Displayed
OvOb	10-1	6 - 11 y	1. Identify children at increased risk for obesity because of parental obesity, change in physical activity +/- excessive gain in BMI for focused CHILD 1 diet/physical activity education<br>2. BMI percentile stable→ reinforce current program, 6 month followup Increasing<br>3. Increasing BMI percentile→ RD counseling for Energy-balanced CHILD 1 diet, intensified physical activity, 3 m followup	1. IF pi_ageInMonths = 72<144 AND pi_hasParentObesity = "Y" AND pi_deltaPhysicalActivity =changed THEN OO10<br>2. IF pi_ageInMonths = 72<144 AND pi_deltaBMI = stable AND pi_bmiPercentile = <85 THEN OO12<br>3. IF pi_ageInMonths = 72<144 AND pi_bmiPercentile = <85 AND pi_deltaBMI = excessiveIncrease OR pi_deltaBMI increasing THEN OO29	1. OO10=<br>Provide focused CHILD1 and physical activity education (B).<br>2. OO12=<br>Reinforce current programs. Follow up in 6 months (B).<br>3. OO29=<br>RD counseling for energy balanced weight control diet, intensified physical activity, follow up in three months (B).

Domain	Guideline Table	Age Range from Guideline	Text From Guidelines	CDS Rule	Recommendation Displayed
OvOb	10-1	6 - 11 y	4. BMI 85th-95th percentile: Excessive weight gain prevention with parents as focus for energy-balanced diet; reinforce physical activity recommendations 6 month followup<br>5. Stable/improving BMI percentile→ reinforce current program, 6 m follow-up<br>6. Increasing BMI percentile→ RD counseling for energy-balanced CHILD 1 diet, intensified physical activity recommendations, 3 month followup	1. IF pi_ageInMonths = 72<144 AND pi_bmiPercentile = 85<95 AND pi_deltaBMI ≠ increasing AND pi_deltaBMI ≠ improving AND pi_deltaBMI ≠ excessiveIncrease AND pi_deltaBMI ≠ stable THEN OO14<br>2. IF pi_ageInMonths = 72<144 AND pi_bmiPercentile = 85<95 AND pi_deltaBMI = improving OR pi_deltaBMI = Stable THEN OO15<br>3. IF pi_ageInMonths= 72<144 AND pi_bmiPercentile= 85<95 AND pi_deltaBMI= excessiveIncrease OR pi_deltaBMI = increasing THEN OO16	1. OO14=<br>Excessive weight gain prevention with parents as focus for energy balanced diet; reinforce physical activity recommendations, follow up in 6 months (D)<br>2. OO15=<br>Reinforce current program, follow up in 6 months (D).<br>3. OO16=<br>RD counseling for energy-balanced CHILD 1 diet, intensified physical activity recommendations, follow up in three months (D).

Domain	Guideline Table	Age Range from Guideline	Text From Guidelines	CDS Rule	Recommendation Displayed
OvOb	10-1	6 - 11 y	1. BMI ≥ 95th percentile: Specific assessment for comorbidities.*	1. If pi_ageInMonths = 72<144 AND pi_bmiPercentile = 95<97 THEN OO17	1. OO17= Assess for comorbidities (B).

Domain	Guideline Table	Age Range from Guideline	Text From Guidelines	CDS Rule	Recommendation Displayed
OvOb	10-1	6 - 11 y	1. BMI ≥ 95th percentile with no comorbidities: Office-based weight loss plan: Family-centered program with parents as focus for behavior modification, (-) energy balance diet counseling by RD, Rx for increased MVPA, decreased sedentary time x 6 months<br>2. Improvement in BMI percentile/comorbidities→ continue current plan<br>3. No improvement in BMI percentile→ referral to comprehensive multidisciplinary lifestyle weight loss program	1. IF pi_ageInMonths=72<144 AND pi_bmiPercentile =95<97 AND "Y" THEN OO18<br>2. IF pi_ageInMonths = 72<144 AND pi_bmiPercentile= 95<97 AND pi_dyslipidemia ="Y" OR pi_hypertension ="Y" OR pi_diabetes2 = "Y" OR pi_deltaBMI = Improving OR ing OR pi_deltaHypertension = improving OR pi_deltaDiabetes2 = improving OR pi_deltaDiabetes1= Improving THEN 0019<br>3. IF pi_ageInMonths = 72<144 AND pi_bmiPercentile = 95<97 AND pi_noCondition = "Y" AND pi_deltaBMI=noImprovement THEN OO20	1. OO18=<br>Office-based weight loss plan. Family-centered program with parents as focus for behavior modification, (-) energy balance diet counseling by RD, Rx for increased moderate to vigorous physical activity, decreased sedentary time for 6 months (A)<br><br>2. OO19= Continue current plan (A).<br>3. OO20= Refer to comprehensive multidisciplinary lifestyle weight loss program (A).

Domain	Guideline Table	Age Range from Guideline	Text From Guidelines	CDS Rule	Recommendation Displayed
OvOb	10-1	6 - 11 y	1. BMI ≥ 95th %ile with co-morbidities, BMI > 97th percentile, or progressive rise in BMI despite therapy: Refer to comprehensive multidisciplinary weight loss program for intensive management x 6 months<br>2. Improvement in BMI percentile→ continue present program<br>3. No improvement in BMI percentile→ consider referral to another comprehensive multidisciplinary weight loss program	1. IF pi_ageInMonths= 72<144 AND pi_bmiPercentile = 95<97 AND pi_dyslipidemia = “Y” OR pi_hypertension = “Y” OR pi_diabetes2 = “Y” THEN 0021<br>OR<br>IF pi_ageInMonths = 72<144 AND pi_bmiPercentile= >=97 THEN OO21<br>2. IF pi_ageInMonths= 72<144 AND pi_bmiPercentile = 95<97 AND pi_dyslipidemia = “Y” OR pi_hypertension = “Y” OR pi_diabetes2 = “Y” AND pi_deltaBMI = Improving THEN OO19<br>3. IF pi_ageInMonths= 72<144 AND pi_bmiPercentile = 95<97 AND pi_dyslipidemia = “Y” OR pi_hypertension = “Y” OR pi_diabetes2 = “Y” AND pi_deltaBMI = noImprovement THEN OO46	4. OO21= Refer to comprehensive multidisciplinary weight loss program for intensive management for 6 months (A).<br>5. OO19= Continue current plan (A).<br>6. OO46= Consider referral to another comprehensive multidisciplinary weight loss program.

Domain	Guideline Table	Age Range from Guideline	Text From Guidelines	CDS Rule	Recommendation Displayed
OvOb	10-1	12 - 21 y	1. Identify adolescents at increased risk for obesity because of parental obesity, change in physical activity +/- excess gain in BMI for focused diet/physical activity education x 6 months<br>2. BMI/BMI percentile stable → reinforce current program, 6 months followup<br>3. Increasing BMI/BMI percentile→ RD counseling for energy-balanced CHILD 1 diet, intensified physical activity x 3 months	1. IF pi_ageInMonths= 144<264 AND pi_hasParentObesity = "Y"= pi_deltaPhysicalActivity= Changed THEN OO24<br>2. IF pi_ageInMonths = 144<264 AND pi_deltaBMI = stable AND pi_bmiPercentile = <85 THEN OO15<br>3. IF pi_ageInMonths = 144<264 AND pi_bmiPercentile = <85 AND pi_deltaBMI = ExcessiveIncrease OR pi_deltaBMI = increasing THEN OO27	1. OO24= Provide focused diet and physical activity education for 6 months (B).<br>2. OO15= Reinforce current program, follow up in 6 months (D).<br>3. OO27= RD counseling for energy-balanced CHILD 1 diet, intensified physical activity for 3 months

Domain	Guideline Table	Age Range from Guideline	Text From Guidelines	CDS Rule	Recommendation Displayed
OvOb	10-1	12 - 21 y	1. BMI 85th-95th percentile: Excess weight gain prevention with adolescent as change agent for energy-balanced CHILD 1 diet, reinforced physical activity recommendations x 6 months<br>2. Improvement in BMI percentile→ continue current program<br>3. Increasing BMI percentile→ RD counseling for energy-balanced weight control diet, intensified physical activity, 3 month followup	1. IF pi_ageInMonths= 144<264 AND pi_bmiPercentile = 85<95 AND pi_deltaBMI≠ Increasing AND pi_deltaBMI ≠ Improving AND pi_deltaBMI ≠ excessiveIncrease THEN OO45<br>2. IF pi_ageInMonths = 144<264 AND pi_bmiPercentile = 85<95 AND pi_deltaBMI = improving THEN OO28<br>3. IF pi_ageInMonths = 144<264 AND pi_bmiPercentile = 85<95 AND pi_deltaBMI = excessiveIncrease OR pi_deltaBMI = increasing THEN OO30	1. OO45= Discuss excess weight gain prevention with adolescent as change agent for energy-balanced CHILD 1 diet, reinforced physical activity recommendations every 6 months<br>2. OO28= Continue current program (B).<br>3. OO30= Assess for comorbidities.
OvOb	10-1	12 - 21 y	1. BMI ≥ 95th percentile: Specific assessment for comorbidities*:	1. IF pi_ageInMonths = 144<264 AND pi_bmiPercentile = >=95 THEN OO30	1. OO30= Assess for comorbidities.

Domain	Guideline Table	Age Range from Guideline	Text From Guidelines	CDS Rule	Recommendation Displayed
OvOb	10-1	12 - 21 y	1. BMI ≥ 95th percentile with no comorbidities: Office-based weight loss plan: Family-centered with adolescent as change agent for behavior modification counseling, RD counseling for (-) energy-balanced diet, Rx for increased MVPA, decreased sedentary time x 6 months<br>2. Improvement in BMI/BMI percentile → continue current program<br>3. No improvement in BMI/ BMI percentile → referral to comprehensive multidisciplinary weight loss program with peers<br>No improvement in BMI/BMI percentile→ consider initiation of medication (orlistat) under care of experienced MD x 6-12 months	1. IF pi_ageInMonths = 144<264 AND pi_bmiPercentile = >=95 AND pi_noCondition =“Y” THEN OO31<br>2. IF pi_ageInMonths = 144<264 AND pi_bmiPercentile = >=95 AND pi_noCondition = “Y” AND pi_deltaBMI = Improving THEN OO32<br>3. IF pi_ageInMonths = 144<264 AND pi_bmiPercentile= >=95 AND pi_noCondition = “Y” AND pi_deltaBMI = NoImprovement THEN OO33	1. OO31=Office-based weight loss plan. Family-centered with adolescent as change agent for behavior modification counseling, RD counseling for (-) energy-balanced diet, Rx for increased moderate to vigorous physical activity, decreased sedentary time x 6 months (B).<br>2. OO32=Continue current program (B).<br>3. OO33= Referral to comprehensive multidisciplinary weight loss program with peers. Consider initiation of medication (orlistat) under care of experienced MD for 6-12 months (B)

Domain	Guideline Table	Age Range from Guideline	Text From Guidelines	CDS Rule	Recommendation Displayed
			1. BMI ≥ 95th %ile with comorbidities or BMI > 35 kg/m^2:<br>Refer to comprehensive lifestyle weight loss program for intensive management x 6-12 months<br>2. Improvement in BMI/BMI percentile→ continue present program<br>3. No improvement in BMI/ BMI percentile→ consider initiation of orlistat under care of	1. IF pi_ageInMonths = 144<264 AND pi_bmiPercentile= >=95 OR pi_dyslipidemia = “Y” OR pi_hypertension =“Y” OR pi_diabetes2= “Y” OR pi_bmi = >=35 AND pi_deltaBMI ≠ improving AND pi_deltaBMI ≠ noImprovement THEN OO34<br>2. pi_ageInMonths = 144<264 AND pi_bmiPercentile = >=95 AND pi_dyslipidemia = “Y” OR pi_hypertension =“Y” OR pi_diabetes2 = “Y” AND pi_deltaBMI = NoImprovement THEN 0035<br>3. pi_ageInMonths = 144<264 AND pi_bmiPercentile = >=95 AND pi_dyslipidemia = “Y” OR pi_hypertension =“Y” OR pi_diabetes2 = “Y” AND pi_deltaBMI = improving THEN 0036	1. OO34= Refer to comprehensive multidisciplinary weight loss program for intensive management for 6 to 12 months (A).<br>2. OO35= Consider initiation of orlistat under care of experienced clinician for 6-12 months (A).

Domain	Guideline Table	Age Range from Guideline	Text From Guidelines	CDS Rule	Recommendation Displayed
FHx	4-1	Birth - 18 y	1. Take detailed family history (FHx) of CVD[a] at initial encounter and/or at 3y, 9-11y & 18 y members	1. IF pi_ageInMonths =0<216 THEN FH1	1. Take detailed family history of CVD at initial encounter and update at the 3, 9-11, and 18 year visits (B).
FHx	4-1	Birth - 18 y	1. If (+) FHx identified, evaluate patient for other CV risk factors, including dyslipidemia, hypertension, diabetes, obesity, history of smoking, and sedentary lifestyle	1. IF pi_ageInMonths = 0<216 AND pi_hasFemaleMaleCVD =“Y” THEN FH2	1. Evaluate patient for other CV risk factors (B).
FHx	4-1	Birth - 18 y	1. If (+) FHx and/or CV risk factors identified, evaluate family, especially parents, for CV risk factors	1. IF pi_ageInMonths= 0<216 AND pi_hasFemaleMaleCVD= “Y” OR pi_dyslipidemia= “Y” OR pi_hypertension = “Y” OR pi_diabetes1= “Y” OR pi_diabetes2= “Y” OR pi_bmiPercentile >=95 OR pi_isSmoker= OR pi_physicalActivity = sedentary THEN FH3	1. Evaluate family, especially parents, for CV risk factors (B).
FHx	4-1	Birth - 18 y	1. Update FHx at each non-urgent health encounter	1. IF pi_ageInMonths = 0<216 THEN FH4	1. Update family history at each non urgent visit (D).

Domain	Guideline Table	Age Range from Guideline	Text From Guidelines	CDS Rule	Recommendation Displayed
FHx	4-1	Birth - 18 y	1. Use FHx to stratify risk for CVD risk as risk profile evolves	1. IF pi_ageInMonths = 0<216 THEN FH5	1. Use family history to stratify risk for CVD as the risk profile evolves (D).
FHx	4-1	18 - 21 y	1. Review FHx of heart disease with young adult patient	1. IF pi_ageInMonths = 216 <264 THEN FH6	1. Review family history of CVD with patient (B).
ND	5-1	Birth - 6 m	1. Infants should be exclusively breast fed (no supplemental formula or other foods) until age 6 m.	1. IF pi_ageInMonths = 0<6 THEN ND1	1. Infants should be exclusively breastfed, no supplemental formula or other foods, until age 6 months (B).

Domain	Guideline Table	Age Range from Guideline	Text From Guidelines	CDS Rule	Recommendation Displayed
ND	5-1	6 - 12 m	1. Continue breast-feeding until at least age 12 m while gradually adding solids; transition to iron-fortified formula until 12 m if reducing breast-feeding. Fat intake in infants less than 12 months of age should not be restricted without medical indication. Limit other drinks to 100% fruit juice < 4 oz/d; No sweetened beverages; encourage water.	1. IF pi_ageInMonths = 6<12 THEN ND2	1. Continue breastfeeding until 12 months while gradually adding solids. Transition to iron-fortified formula until 12 months if reducing breastfeeding (B). Fat intake in infants less than 12 months of age should not be restricted without medical indication. Limit other drinks to less then 4oz per day of 100% fruit juice. No sweetened beverages, and encourage water (D).

Domain	Guideline Table	Age Range from Guideline	Text From Guidelines	CDS Rule	Recommendation Displayed
ND	5-1	12 - 24 months	1. Transition to reduced-fat[b] (2% to fat-free) unflavored cow's milk[c] (see Supportive Actions bullet 1) Limit/avoid sugar-sweetened beverage intake; encourage water. Transition to table food with: Total fat 30% of daily kcal/EERd. Saturated fat 8-10% of daily kcal/EER. Cholesterol<300 mg/d (B). Avoid trans fat as much as possible. Monounsaturated and polyunsaturated fat up to 20% of daily kcal/EER. Cholesterol < 300 mg/d	1. IF pi_ageInMonths = 12<24 THEN ND3	1. Transition to reduced fat unflavored cow's milk, limit or avoid sugar sweetened beverage intake, and encourage water(B). | Transition to table food with: Total fat 30% of daily kcal/EERd. Saturated fat 8-10% of daily kcal/EER. Cholesterol<300 mg/d (B). Avoid trans fat as much as possible. Monounsaturated and polyunsaturated fat up to 20% of daily kcal/EER. (D).

<table>
<tr><th>Domain</th><th>Guideline Table</th><th>Age Range from Guideline</th><th>Text From Guidelines</th><th>CDS Rule</th><th>Recommendation Displayed</th></tr>
<tr><td>ND</td><td>5-1</td><td>2 -10 years</td><td>1. Primary beverage: Fat-free unflavored milk. Limit/avoid sugar sweetened beverages; encourage water. Fat content: Total fat 25-30% of daily kcal/EER[d.] Saturated fat 8-10% of daily kcal/ EER. Avoid trans fats as much as possible. Monounsaturated and polyunsaturated fat up to 20% of daily kcal/EER. Cholesterol < 300 mg/d. Encourage high dietary fiber intake from foods</td><td>1. IF pi_ageInMonths = 24<132 THEN ND4</td><td>1. Fat free unflavored milk (A), limit or avoid sugar sweetened beverages; encourage water (B). | Total fat 25-30% of daily kcal/EERd, Saturated fat 8-10% of daily kcal/ EER, Cholesterol<300 mg/d (A). Avoid trans fats as much as possible Monounsaturated and polyunsaturated fat up to 20% of daily kcal/EER, (D). | Encourage high dietary fiber intake from foods (B).</td></tr>
</table>

Domain	Guideline Table	Age Range from Guideline	Text From Guidelines	CDS Rule	Recommendation Displayed
ND	5-1	11 - 21 y	1. Primary beverage: Fat-free unflavored milk. Limit/ avoid sugar sweetened beverages; encourage water. Fat content: Total fat 25-30% of daily kcal/EER[d]. Saturated fat 8-10% of daily kcal/ EER. Avoid trans fat as much as possible. Monounsaturated and polyunsaturated fat up to 20% Grade D of daily kcal/ EERCholesterol < 300 mg/d, Encourage high dietary fiber intake from foods	1. IF pi_ageInMonths = 132<252 THEN ND5	1. Fat free unflavored milk (A), limit or avoid sugar sweetened beverages; encourage water (B). Total fat 25-30% of daily kcal/EERd, Saturated fat 8-10% of daily kcal/ EER, Cholesterol<300 mg/d (A). Avoid trans fats as much as possible Monounsaturated and polyunsaturated fat up to 20% of daily kcal/EER, (D). Encourage high dietary fiber intake from foods (B).
PA	6-1	0-12 m	1. Parents should create an environment promoting and modeling physical activity and limiting sedentary time	1. IF pi_ageInMonths = 0<12 THEN PA1	1. Parents should create an environment promoting and modeling physical activity and limiting sedentary time (D).

Domain	Guideline Table	Age Range from Guideline	Text From Guidelines	CDS Rule	Recommendation Displayed
PA	6-1	1- 4 y	1. Unlimited active playtime in safe, supportive environment. Limit sedentary time, especially TV/ video	1. IF pi_ageInMonths = 12<60 THEN PA2	1. Unlimited active playtime in safe, supportive environment. Limit sedentary time, especially TV and video (D).
PA	6-1	5 - 10 y	1. Moderate to vigorous physical activity* every day. Limit daily leisure screen time (TV/video/computer)	1. IF pi_ageInMonths = 60<132 THEN PA3	1. Moderate to vigorous physical activity every day (A). Limit daily leisure screen time- TV/video/computer (B).
PA	6-1	11 -17 y	1. Moderate to vigorous physical activity* every day. Limit leisure time TV/video/computer use.	1. IF pi_ageInMonths = 132<216 THEN PA4	1. Moderate to vigorous physical activity every day (A). Limit leisure time TV/video/computer use(B).
PA	6-1	18 - 21 y	1. Moderate to vigorous physical activity* every day. Limit leisure time TV/video/computer.	1. IF pi_ageInMonths = 216<262 THEN PA5	1. Moderate to vigorous physical activity every day (A). Limit leisure time TV/video/computer use (B).

Domain	Guideline Table	Age Range from Guideline	Text From Guidelines	CDS Rule	Recommendation Displayed
TE	7-1	0 -12m<br>1 - 4 y	1. Smoke free home environment. Reinforce this message at every encounter, including urgent visits for respiratory problems	1. IF pi_ageInMonths = 0<60 THEN TE1	1. Promote a smoke free home environment (B) . Reinforce this message at every encounter, including urgent visits for respiratory problems (C).
TE	7-1	5 - 10 y	1. Obtain smoke exposure history from child, including personal history of tobacco use. Counsel patients strongly about not smoking, including providing explicit information about the addictive and adverse health effects of smoking	1. IF pi_ageInMonths = 60<132 THEN TE2	1. Obtain smoke exposure history from child, including personal history of tobacco use. Counsel patients strongly about not smoking, including providing explicit information about the addictive and adverse health effects of smoking (C).

Domain	Guideline Table	Age Range from Guideline	Text From Guidelines	CDS Rule	Recommendation Displayed
TE	7-1	11 - 17 y<br>18 - 21 y	1. Obtain personal smoking history at every non-urgent health encounter. Explicitly recommend against smoking.<br>2. Provide specific smoking cessation guidance	1. IF pi_ageInMonths = 132<252 THEN TE3<br>2. IF pi_ageInMonths = 132<252 AND pi_isSmoker =“Y” THEN TE4	1. Obtain personal smoking history at every non-urgent health encounter. Explicitly recommend against smoking (B)<br>2. Provide specific smoking cessation guidance (B).
LP	9-5	Birth–2 years	1. No lipid screening	1. IF pi_ageInMonths= 0<24 THEN LP58	1. No lipid screening (C)
LP	9-5	2–8 years	1. No routine lipid screening	1. IF pi_ageInMonths =24<108 AND pi_parent_TC ≠ >=240 AND pi_parent_Dyslipidemia ≠ “Y” AND pi_hasFemaleMaleCVD ≠ “Y” AND pi_hasOtherRiskCondition ≠ “Y” AND pi_hypertension ≠ “Y” AND pi_isSmoker ≠ “Y” AND pi_bmiPercentile ≠ >=95 AND pi_diabetes1 ≠ “Y” AND pi_diabetes2 ≠ “Y” THEN LP59	1. LP59= No Routine lipid screening (B)

Domain	Guideline Table	Age Range from Guideline	Text From Guidelines	CDS Rule	Recommendation Displayed
LP	9-5	2–8 years	Measure fasting lipid profile (FLP) × 2a; average results if:<br>1. Parent, grandparent, aunt/uncle, or sibling with myocardial infarction (MI), angina, stroke, coronary artery bypass graft (CABG)/stent/angioplasty at <55 years in males, <65 years in females<br>2. Parent with TC ≥240 mg/dL<br>3. or known dyslipidemia<br>4. Child has diabetes, hypertension, BMI ≥95th percentile or smokes cigarettes. Child has a moderate- or high-risk medical condition (Table 9-7)	1. IF pi_ageInMonths = 24<108 AND pi_hasFemaleMaleCVD = "Y" THEN LP1<br>2. IF pi_ageInMonths = 24<108 AND pi_parent_TC = >=240 THEN LP1<br>3. IF pi_ageInMonths = 24<108 AND pi_parent_Dyslipidemia ="Y" THEN LP1<br>4. IF pi_ageInMonths = 24<108 AND pi_hasOtherRiskCondition = "Y" OR pi_hypertension = "Y" OR pi_isSmoker = "Y" OR pi_bmiPercentile = >=95 OR pi_diabetes1 = "Y" OR pi_diabetes2 = "Y" THEN LP1	1. LP1= Measure fasting lipid profile two times and average results (B).

Domain	Guideline Table	Age Range from Guideline	Text From Guidelines	CDS Rule	Recommendation Displayed
LP	9-5	9-11 years	Universal Screening<br><br>1. Non-FLP: Calculate non-HDL-C: Non HDL C = TC - HDL C[c]<br>2. Non-HDL ≥145 mg/dL,<br>3. HDL< 40 mg/dL →FLP × 2, lipid algorithms below[d] OR<br>4. FLP: LDL-C ≥130 mg/dL,<br>5. non-HDL-C ≥145 mg/dL<br>6. HDL-C <40 mg/dL,<br>7. TG ≥100 mg/dL if < 10 years;<br>8. ≥130 mg/dL if ≥10 years → Repeat FLP after 2 weeks but within 3 months → lipid algorithms below[d]	1. IF pi_ageInMonths =108<144 THEN LP10<br>2. IF pi_ageInMonths =108<144 AND pi_NonHDLC_av >=145 THEN LP1<br>3. IF pi_ageInMonths =108<144 AND pi_HDLC_av <40 THEN LP1<br>4. IF pi_ageInMonths =108<144 AND pi_ldlC_av >=130 THEN LP1<br>5. IF pi_ageInMonths =108<144 AND pi_NonHDLC_av = >=145 THEN LP1<br>6. IF pi_ageInMonths =108<144 AND pi_HDLC_av = <40 THEN LP1<br>7. IF pi_ageInMonths = 108 <120 AND pi_tg_av >=100 THEN LP1<br>8. IF pi_ageInMonths = 120<144 AND pi_tg_av >=130 THEN LP1	1. LP10= Measure non fasting lipid profile and calculate non-HDL-C , or measure fasting lipid profile (B).<br>2. THROUGH 8. LP1= Measure fasting lipid profile two times and average results (B

Domain	Guideline Table	Age Range from Guideline	Text From Guidelines	CDS Rule	Recommendation Displayed
LP	9-5	12-16 years	1. No routine screening	1. IF pi_ageInMonths =24<108 AND pi_parent_TC ≠ >=240 AND pi_parent_Dyslipidemia ≠ “Y” AND pi_hasFemaleMaleCVD ≠ “Y” AND pi_hasOtherRiskCondition ≠ “Y” AND pi_hypertension ≠ “Y” AND pi_isSmoker ≠ “Y” AND pi_bmiPercentile ≠ >=85 AND pi_diabetes1 ≠ “Y” AND pi_diabetes2 ≠ “Y” THEN LP59	1. LP59= No Routine lipid screening (B)

Domain	Guideline Table	Age Range from Guideline	Text From Guidelines	CDS Rule	Recommendation Displayed
LP	9-5	12-16 years	Measure FLP × 2[f], average results, if new knowledge of:<br>1. Parent, grandparent, aunt/uncle or sibling with MI, angina, stroke, CABG/ stent/angioplasty, sudden death at < 55 years in males, < 65 years in females<br>2. Parent with TC ≥240 mg/dL or<br>3. known dyslipidemia<br>4. Patient has diabetes, hypertension, BMI ≥85th percentile or smokes cigarettes Patient has a moderate- or high-risk medical condition (Table 9–7)	1. IF pi_ageInMonths = 144<204 AND pi_hasFemaleMaleCVD = "Y" THEN LP1<br>2. IF pi_ageInMonths =144<204 AND pi_parent_TC = >=240 THEN LP1<br>3. IF pi_ageInMonths = 144<204 AND pi_parent_Dyslipidemia = "Y" THEN LP1<br>4. IF pi_ageInMonths = 144<204 AND pi_hasOtherRiskCondition = "Y" OR pi_hypertension = "Y" OR pi_isSmoker = "Y" OR pi_bmiPercentile = >=85 OR pi_diabetes1 = "Y" OR pi_diabetes2 = "Y" THEN LP1	1. LP1= Measure fasting lipid profile two times and average results (B).

Domain	Guideline Table	Age Range from Guideline	Text From Guidelines	CDS Rule	Recommendation Displayed
LP	9-5	17-21 years	1. Universal screening once in this time period: Non-FLP: Calculate non-HDL–C: Non-HDL–C = TC − HDL–C[g]<br>2. 17–19 years: Non-HDL–C ≥145 mg/dL,<br>3. HDL–C<40 mg/dL →FLP × 2, lipid algorithm below (Figure 9–1)OR FLP:<br>4. LDL–C ≥130 mg/dL,<br>5. non-HDL–C ≥145 mg/dL<br>6. HDL–C < 40 mg/dL,<br>7. TG ≥130 mg/dL → Repeat FLP after 2 weeks but within 3 months→ lipid algorithms in Figures 9–1 and 9–2.	1. IF pi_ageInMonths =204<264 THEN LP10<br>2. IF pi_ageInMonths =204<240 AND pi_NonHDLC_av >=145 THEN LP1<br>3. IF pi_ageInMonths =204<240 AND pi_HDLC_av <40 THEN LP1<br>4. IF pi_ageInMonths =204<240 AND pi_ldlC_av >=130 THEN LP1<br>5. IF pi_ageInMonths =204<240 AND pi_NonHDLC_av = >=145 THEN LP1<br>6. IF pi_ageInMonths =204<240 AND pi_HDLC_av = <40 THEN LP1<br>7. IF pi_ageInMonths = 204<240 AND pi_tg_av >=130 THEN LP1	1. LP10= Measure non fasting lipid profile and calculate non-HDL-C , or measure fasting lipid profile (B).<br>2. THROUGH 7.<br>LP1= Measure fasting lipid profile two times and average results (B

Domain	Guideline Table	Age Range from Guideline	Text From Guidelines	CDS Rule	Recommendation Displayed
LP	9-5	17-21 years	20–21 years<br>1. Non-HDL–C ≥190 mg/dL,<br>2. HDL–C < 40 mg/dL[h]<br>→ FLP × 2[i] average results → Adult Treatment Panel III (ATP III) management algorithm OR<br>3. FLP: LDL–C ≥160 mg/dL,<br>4. non-HDL–C ≥190 mg/dL<br>5. HDL–C <40 mg/dL,<br>6. TG ≥150 mg/dL → Repeat FLP after 2 weeks but within 3 months, average results → ATP III management algorithm	1. IF pi_ageInMonths =240<264 AND pi_NonHDLC_av >=190 THEN LP70<br>2. IF pi_ageInMonths =240<264 AND pi_HDLC_av <40 THEN LP70<br>3. IF pi_ageInMonths =240<264 AND pi_ldlC_av >=160 then LP70<br>4. IF pi_ageInMonths =240<264 AND pi_NonHDLC_av >=190 THEN LP70<br>5. IF pi_ageInMonths =240<264 AND pi_HDLC_av <40 THEN LP70<br>6. IF pi_ageInMonths = 240<264 AND pi_tg_av >=150 THEN LP70	1. LP70= Measure fasting lipid profile two times. Adult Treatment Panel III (ATP III) management algorithm) (B).

Domain	Guideline Table	Age Range from Guideline	Text From Guidelines	CDS Rule	Recommendation Displayed
BP	8-1	Birth to 3 y	1. No routine BP measurement<br><br>Measure BP if history (+) for neonatal complications, congenital heart disease, urinary/ renal abnormality, solid-organ transplant, malignancy, drug Rx, or condition known to raise BP or increase intracranial pressure (Table 8-2)<br>2. If BP > 90th %ile by oscillometry, confirm by auscultation<br>→If BP confirmed ≥ 90th %ile, initiate evaluation for etiology and treatment per algorithm. (Figure 8-2)	1. IF pi_ageInMonths 0<36 THEN BP1<br>2. IF pi_ageInMonths 0<36 AND pi_bpSysPercentile_av >=90 AND pi_bpDiaPercentile_av >=90 AND pi_measureTypeBP ≠ Auscultation THEN BP40	1. BP1=<br>No routine BP measurment.<br>Measure BP if history (+) for neonatal complications, congenital heart disease, urinary/ renal abnormality, solid-organ transplant, malignancy, drug Rx, or condition known to raise BP or increase intracranial pressure (D).<br>2. BP40=<br>Confirm by auscultation. (D)

Domain	Guideline Table	Age Range from Guideline	Text From Guidelines	CDS Rule	Recommendation Displayed
			Annual BP measurement in all, interpreted for age/sex/height per Tables 8-3 and 8-4 below<br><br>1. BP < 90th %ile, repeat in 1 year<br><br>2. BP ≥ 90th %ile:<br><br>o Repeat BP X 2 by auscultation<br><br>o Average replicate measurements → Re-evaluate BP category<br><br>→If BP confirmed ≥ 90th %ile, < 95th %ile = Prehypertension (HTN)<br><br>o Recommend weight management if indicated<br><br>o Repeat BP in 6 months<br><br>→If BP ≥ 95th %ile, < 99th %ile + 5mmHg<br><br>o Repeat BP in 1-2 weeks, average all BP measurements"		

Domain	Guideline Table	Age Range from Guideline	Text From Guidelines	CDS Rule	Recommendation Displayed
			Annual BP measurement in all, interpreted for age/sex/height per Tables 8-3 and 8-4 below<br><br>**1.** BP < 90th %ile, counsel on CHILD 1 diet, activity recommendations, and repeat BP in 1 year<br><br>**2.** BP ≥ 90th %ile or > 120/80:<br><br>o Repeat BP X 2 by auscultation<br><br>o Average replicate measurements → Re-evaluate BP category<br><br>→If BP confirmed ≥ 90th %ile, < 95th %ile or ≥ 120/80 = Pre-HTN<br><br>o CHILD 1 diet, activity recommendations, weight management if indicated<br><br>o Repeat BP in 6 months<br><br>→If BP ≥ 95th %ile, < 99th %ile + 5mmHg		

Domain	Guideline Table	Age Range from Guideline	Text From Guidelines	CDS Rule	Recommendation Displayed
BP	8-1	18 - 21 y	1. Measure BP at all health care visits<br>2. BP ≥ 120/80 to 139/89 = Pre-HTN<br>3. BP ≥ 140/90 to 159/99 = Stage 1 HTN<br>4. BP ≥ 160/100 = Stage 2 HTN<br><br>Evaluation/ Treatment per JNC recommendations	1. IF pi_ageInMonths = 216<264 AND pi_bpSys <120 AND pi_bpDia <80 THEN BP20<br>2. IF pi_ageInMonths = 216<264 AND pi_bpSys 120<140 AND pi_bpDia 80<90 THEN BP21<br>3. IF pi_ageInMonths = 216<264 AND pi_bpSys 140<160 AND pi_bpDia 90<100 THEN BP22<br>4. IF pi_ageInMonths = 216<264 AND pi_bpSys >=160 AND pi_bpDia >=100 THEN BP23	1. BP20= Measure BP at all health care visits.<br>2. BP21= Pre-Hypertension. Evaluation and treatment per JNC recommendations.<br>3. BP22= Stage 1 Hypertension. Evaluation and treatment per JNC recommendations.<br>4. BP23= Stage 2 Hypertension. Evaluation and treatment per JNC recommendations.
